# Editorial: New insights into the role of mycobiome in diseases

**DOI:** 10.3389/fcimb.2023.1240657

**Published:** 2023-06-30

**Authors:** Wenjuan Wu, Jun Ding, Xiaoqi Zheng, Hui Wang, Ning-Ning Liu

**Affiliations:** ^1^ Department of Laboratory Medicine, Shanghai East Hospital, Tongji University School of Medicine, Shanghai, China; ^2^ Meakins-Christie Laboratories, Department of Medicine, McGill University Health Centre, Montreal, QC, Canada; ^3^ State Key Laboratory of Systems Medicine for Cancer, Center for Single-Cell Omics, School of Public Health, Shanghai Jiao Tong University School of Medicine, Shanghai, China

**Keywords:** mycobiome, fungi, interaction, multi-omic, diseases

Fungi, widely distributed in nature, play important roles in maintenance of ecological biodiversity. Although constitutes for less than 0.1% of the total gut microbiome, mycobiome, an essential component of the microbiome, has been reported to be closely related with human diseases recently (VanEvery et al., 2023). The human bodies are colonized with diverse fungi, which impose significant effects on human health by direct or indirect interactions with the host barrier function, other microbiota components (Liu et al., 2022), and immune system. In addition, it can also function as a reservoir of pathogenic fungi when the host immune system is compromised or during the progression of inflammatory diseases, metabolic disorders and cancers. Therefore, this Research Topic aims to discuss the role and effect of mycobiome in various diseases from new perspectives (Saftien et al., 2023).

Extensive studies have explored the interactions among mycobiome, immune cells and cellular metabolisms. Yu et al. found that the fungi depletion by antibiotic treatment induced higher expression of the hub enzymes and transporters involved in CD4^+^ T cell glutaminolysis (Yu et al.). Moreover, they discovered that fungi could activate the dectin-1-Syk- NF-kB signaling pathway to elevate the oxidative phosphorylation (OXPHOS) so that they could promote the pathogenesis of inflammatory bowel disease (IBD). Therefore, fungi could directly increase the production of pro-inflammatory cytokines, affect the host immune response, regulate glutaminolysis to affect the CD4^+^ T cell differentiation, and influence the expression of inflammation-related gene. These results support that mycobiome is closely correlated with reprogramming of the immune cell metabolism. In addition, ferroptosis, a non-apoptotic and iron-dependent form of nonapoptotic cell death, is intrinsically associated with diverse cellular metabolic pathways such as cellular respiration, amino acid metabolism, lipid metabolism and so forth. It has also been reported to be involved in the progression of many diseases, including infections, cardiovascular diseases, even cancer and so on. Recently, Teng et al. identified several hub genes and pathways of ferroptosis in *Fusarium* keratitis by combination of a series of bioinformatic methods and verified by qPCR. The potential role of ferroptosis in *Fusarium* keratitis still awaits further investigation, which may provide potential therapeutic insights against *Fusarium* keratitis and other fungal diseases (Teng et al.).

When the host is immunocompromised or its barrier functions are destroyed, the fungal pathogens would invade and infect the host, causing opportunistic infection. *Prototheca wickerhamii*, initially identified as a fungus, is a pathogen causing mild or severe, even fatal protothecosis. Guo et al. investigated the morphological differences and the pathogenic mechanisms of different strains of *Prototheca wickerhamii* through multi-omics data analysis, including transcriptomics, proteomics, and metabolomics, to uncover the possible pathogenesis mechanism (Guo et al.). They observed that the *P. wickerhamii* S1 strain has caused much less damage to macrophages than HN01 with a different morphology. These results highlights that it is urgent to understand the ecology and pathogenesis from a One Health perspective (i.e. the relationship among fungi, humans, environments, animals and plants).

In addition, we should pay more attention to each step of mycobiome analysis such as DNA isolation, primer design and choice, polymerase selection, sequencing platform selection, and fungal reference databases to avoid contamination or incomplete or erroneous sequences. Arfken et al. highlighted the importance of preliminary studies to evaluate primer combinations and database selection for the mycobiome analysis of sample of interest. They assessed the effects of community composition on taxon abundance and raised questions regarding the validity of fungal abundance estimates utilizing a mixed mock community isolated from piglet feces (Arfken et al.).

Taken together, researches mentioned above inspired us to shed light on the effect of mycobiome in diseases from a comprehensive insight. This will not only help us understand the mechanism of mycobiome-host interaction during pathogenesis and disease progression, but also eventually provide potential therapeutic insights.

## Author contributions

All authors listed have made a substantial, direct, and intellectual contribution to the work and approved it for publication.
